# Does a Positive Response to Transforaminal Epidural Steroid Injection Identify Patients Who Can Avoid Surgery for Two Years?

**DOI:** 10.1155/2023/4298436

**Published:** 2023-10-14

**Authors:** Aki Fujiwara, Keisuke Watanabe, Hideki Shigematsu, Katsuhiro Kimoto, Mitsuru Ida, Yasuhito Tanaka, Masahiko Kawaguchi

**Affiliations:** ^1^Department of Anesthesiology, Nara Medical University, Nara 634-8522, Japan; ^2^Department of Orthopaedics, Nara Medical University, Nara 634-8522, Japan

## Abstract

**Background:**

Transforaminal epidural steroid injection (TFESI) is widely used to manage lumbar radiculopathy. In clinical settings, patients often undergo repeated transforaminal epidural injections with or without steroid administration.

**Objectives:**

To examine whether a positive response to TFESI at the first month, can in clinical settings, identify patients with radiculopathy who can avoid surgery for two years. *Study Design/Setting*. This prospective observational study was conducted at an academic medical center.

**Methods:**

Individuals aged ≥20 years who had been referred to our pain center by spine surgeons were enrolled. All patients were assessed using the Numerical Rating Scale (NRS) at baseline and 1 month after the first TFESI. Patients were divided into two groups according to the NRS decrement: the positive response (PR) group achieved a ≥2.0 decrease on the NRS 1 month after the first TFESI compared to baseline and the no response (NR) group achieved a <2.0 decrease on the NRS. The incidence rates of surgery over two years were compared between the two groups. In addition, we calculated the hazard ratio of the PR group to the NR group regarding the incidence of surgery over two years using the Cox proportional hazard model, adjusting for baseline NRS.

**Results:**

Seventy-six patients completed the two-year follow-up. In total, 8 and 68 patients had bilateral and unilateral radiculopathy, respectively. The PR and NR groups included 35 and 41 patients, respectively. The rate of surgery avoidance was 85.7% and 73.2% in the PR and NR groups, respectively. This difference was not statistically significant (*p*=0.26). After adjusting for baseline NRS, the hazard ratio of the PR group to the NR group regarding the incidence of surgery within two years was 0.35 (95% confidence interval: 0.11–1.11, *p*=0.08).

**Conclusion:**

A positive response to TFESI may not identify patients who can avoid surgery for two years.

## 1. Introduction

Lumbar radiculopathy can be caused by lumbar disc herniation or degenerative vertebral changes. Irritation and compression of nerve roots cause inflammation and pain [1]. Therefore, local delivery of corticosteroids with both anti-inflammatory and local anesthetic properties to the affected nerve root is essential for pain relief [2]. The short-term efficacy of transforaminal epidural steroid injection (TFESI) for lumbar radiculopathy has been demonstrated [3, 4]. Several studies have reported reduced rates of surgery following TFESI [5]. In our experience, some patients with radiculopathy can avoid surgery by repeating interventions.

The question of who would benefit from continuing conservative treatment including nerve block remains debatable. Shortening the illness duration and avoiding indiscriminate repeated nerve block treatment by determining patients who require surgical treatment at an early stage is regarded as beneficial for both patients and the medical economy. We hypothesized that a positive response to TFESI at the first month would be useful for identifying patients who could avoid surgical treatment from those who require surgical treatment. This study aimed to investigate whether a positive response to TFESI at the first month was useful to identify patients who could avoid surgery for two years among patients with radiculopathy and examine the surgery-avoidance rate by repeating the intervention for two years.

## 2. Materials and Methods

This prospective observational study was approved by the appropriate Institutional Review Board (approval number, 1235; June 7, 2016) and was jointly conducted by the Department of Anesthesiology and the Department of Orthopedics at our institution.

Of the patients with spinal disorders referred by spine surgeons to our pain center between July 01, 2016, and December 31, 2018, those who fulfilled the following inclusion criteria were eligible to participate in the study: adults aged 20 years and older; presence of lower extremity pain with or without low back pain due to lumbar spinal canal stenosis, herniated disc, or lumbar spondylolisthesis; and symptoms consistent with physical and imaging findings. We included patients with stable symptoms whose etiology was easy to understand. We excluded patients with cauda equina syndrome and bladder and bowel dysfunction; with lower extremity muscle weakness (manual muscle test < 4); without lower extremity pain; and who had a history of lumbar spine surgery, a fresh fracture of the lumbar spine (low signal in *T*1-weighted sequences and high signal on *T*2 on magnetic resonance imaging (MRI)), or a tumor, cyst, or infection in the spine.

In addition, we excluded patients who were unable to undergo fluoroscopic TFESI. We excluded patients with severe renal dysfunction (glomerular filtration rate of < 30 mL/min/1.73 m^2^), uncontrolled diabetes mellitus (hemoglobin A1c level of > 8.0%), thrombocytopenia (platelet count of < 50,000/*μ*L) or bleeding tendency; patients currently taking an antithrombotic; and patients allergic to contrast media or local anesthetics.

Participants who were unable to complete the questionnaire owing to factors such as severe dementia were also excluded. All eligible patients provided written informed consent.

### 2.1. Patient Enrolment

Spine surgeons diagnosed the primary disease in patients with lower extremity pain based on history, physical examination (provocation test, sensory disturbance, manual muscle test, and pain distribution consistent with lumbar radiculopathy), and MRI or computed tomography (CT) scan results documenting disc herniation or degenerative disease with nerve root compression at the level and side of clinical symptoms. The spine surgeons proposed both surgical treatment and nerve blocks for these patients. Patients who requested nerve block treatment were subsequently referred to our pain center.

Pain physicians at the pain center diagnosed their primary disease again based on symptoms, physical examination (provocation test, sensory disturbance, and manual muscle test), and imaging findings (radiography, MRI, and CT). After clarification that their symptom was caused by lumbar radiculopathy, pain physicians specified the affected nerve root based on symptoms and physical examination. Provocation tests included the straight leg raise, femoral nerve stretching, Newton, Patrick, Gaenslen, Freiberg, flexion, adduction, and internal rotation (FAIR) tests. Patients eligible for TFESI were encouraged to participate in the present study.

### 2.2. Interventions

In all patients, unilateral or bilateral TFESI was performed under fluoroscopy at the initial clinical visit to the pain center. After confirming the epidural space with a contrast medium (1-2 mL of iohexol), 2 mL of 0.5% mepivacaine and 1.65 mg of dexamethasone were injected. When performing bilateral TFESI, half of the 2 mL of 0.5% mepivacaine and 1.65 mg of dexamethasone were injected into each side. TFESI was repeated at the same level 2 weeks later, when necessary, depending on the individual patient's symptoms. Patients received TFESI based on their symptoms; if a patient had a herniated disc on L5/S1 compressing the S1 nerve root, TFESI was performed through the S1 dorsal sacral foramen.

After one month, the attending pain physician determined the type and frequency of treatment based on the needs and conditions of the patient. There was no restriction of treatment to the extent of the national insurance system as with normal medical care. Patient follow-up was simultaneously performed by a pain physician and a spine surgeon; the patient could express the desire to undergo surgical treatment to the spine surgeon at any time.

### 2.3. Assessment

After obtaining written informed consent to participate in the study at the initial visit to the pain center, pain intensity was assessed using the Numerical Rating Scale (NRS) from 0 to 10 (0, no pain and 10, maximum pain that can be imagined), health-related quality of life (QOL) was measured using the EuroQOL 5-Dimension 5-Level (EQ5D5L) [6], anxiety and depression were assessed using the Hospital Anxiety and Depression Scale (HADS) [7], and catastrophic thinking was assessed using the Pain Catastrophizing Scale (PCS) [8]. The NRS and EQ5D5L scores were reassessed after 1 month. Treatment (e.g., epidural injection, intravenous steroid administration, orally administered drugs including NSAIDs, tramadol, antiepileptic drugs, and antidepressants) administered before the start of the treatment at the pain center was assessed. Treatment before consultation with the pain center was assessed using information from medical referral letters and interviews.

### 2.4. Grouping

Given that the minimal clinically important difference (MCID) of NRS is reported to be 2.0 [9, 10], patients with a decrease of ≥2.0 in the NRS at 1 month after the initial TFESI were assigned to the positive response (PR) group, and those with a decrease of <2.0 were assigned to the no response (NR) group.

The proportions of patients who underwent surgery within one and two years were compared between the groups. Information pertaining to the surgery was obtained from electronic patient records if the patient continued to visit our hospital due to treatment for other disorders. When the electronic patient records could not be used because of dropout, we made telephone calls to confirm their current situation. In addition, we examined the hazard ratio of the PR group to the NR group regarding the surgery-avoidance rate for two years using the Cox proportional hazards model adjusted for baseline NRS.

### 2.5. Instruments

#### 2.5.1. EQ5D5L

The EQ5D5L is a standard tool used to measure QOL in five dimensions: mobility, self-care, usual activities, pain/discomfort, and anxiety/depression. Each dimension has five levels and together defines 3,125 health states. It has a country-specific scoring system, and in Japan, these health states are expressed as a value from −0.025 to 1 (full health) [6]. The MCID is 0.1 for chronic pain [11].

#### 2.5.2. HADS

The HADS is a self-assessment tool developed to evaluate anxiety and depression and consists of 14 items: seven items for the anxiety subscale (HADS anxiety) and seven for the depression subscale (HADS depression), each scored between 0 and 3. A patient with a total score of 0–7 for each dimension was considered as being asymptomatic, a score of 8–10 indicated mild anxiety or depression symptoms, and a score of 11–21 indicated significant anxiety or depression symptoms [7].

#### 2.5.3. PCS

The PCS consists of 13 items, each of which is rated on a scale of 0 to 4. Points were allocated according to the answer to each item; if the total score was greater than or equal to 30, the level of catastrophic thinking was considered high [8].

### 2.6. Statistical Analysis

A comprehensive review of the published data demonstrated reduced rates of surgery following TFESI. At one year, the rate of surgery avoidance varies between 56% and 90% [5].

In this study, assuming that the positive response rate of TFESI was 50%, the incidence of surgery was estimated to be 20% and 50% in patients in whom TFESI was effective and ineffective, respectively. Based on the calculations of *α* = 0.05 and 1 − *β* = 0.8, the required sample size was *n* = 74. With an estimated dropout rate of 10%, this study aimed to enroll 83 patients. Univariate analysis was performed using the Mann–Whitney *U* test, chi-square independence test, or Fisher's exact test, as appropriate.

A study showed that younger age and unemployment were related to a preference for surgery [12]. Therefore, we examined the hazard ratio of the PR group to the NR group regarding the incidence of surgery over two years using the Cox proportional hazards model, adjusting for baseline NRS.

Statistical analyses were performed using the SPSS software version 23.0 (IBM Corp., Armonk, NY, U.S.A.). Statistical significance was set at *p* < 0.05.

## 3. Results

**Figure 1:**
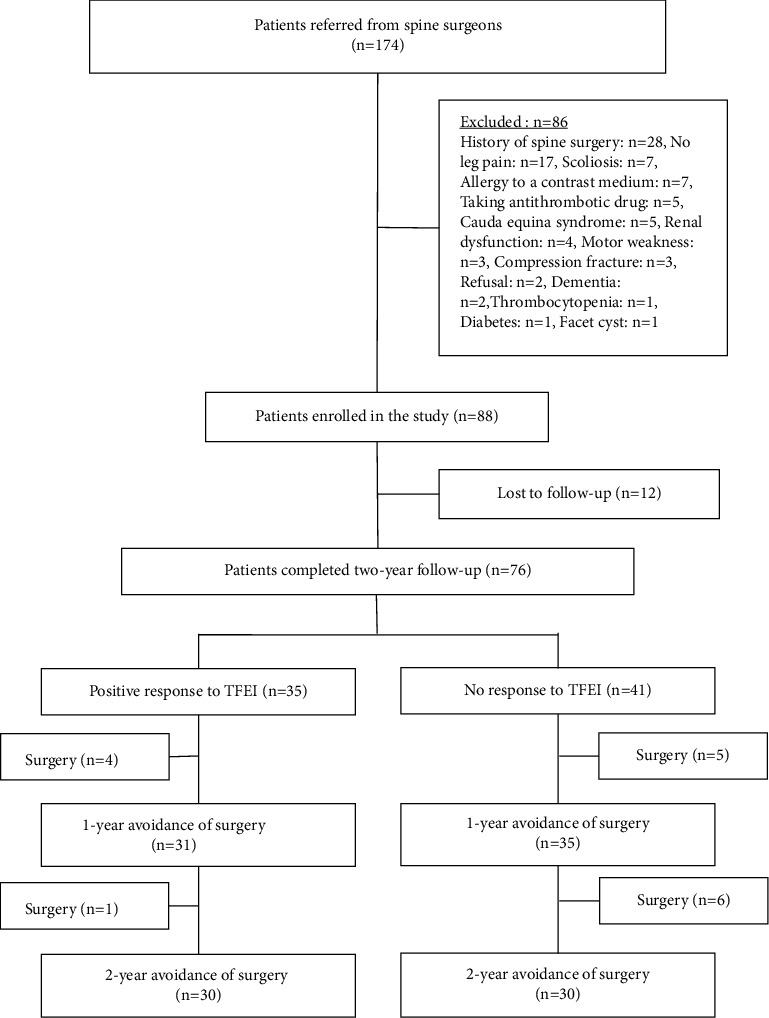
Flow diagram of this study. TFEI: transforaminal epidural injection.

Figure 1 shows the patient flowchart. A total of 88 patients were eligible; however, only 76 patients completed the two-year follow-up. Of the 76 patients, 46.1% (35/76) and 53.9% (41/76) were classified into the PR and NR groups, respectively. There were no significant differences in patient background between the two groups (Table 1). An overview of the primary diseases and treatment levels is shown in Table 2. At the initial visit, the PR group had a significantly higher baseline NRS score than the NR group (*p* < 0.01). The EQ5D5L, HADS anxiety, HADS depression, and PCS scores showed no significant differences between the two groups. Regarding the proportion of patients who continued to visit our pain center, there was no significant difference between the groups (*p*=0.10 for 1 year and *p*=0.25 for 2 years). Details of the interventions are shown in Table 3. Of the 31 patients who continued to visit our pain center, none had a newly emerging symptom in the lumbosacral spine area. Nine of them underwent a new MRI within two years. One of the patients showed a progression of spinal stenosis at the same level, and another showed a reduction of the herniated disc. The other seven patients showed no significant changes. There was no significant difference between the groups in the total number of interventions, including epidural injection (transforaminal/interlaminar/caudal), pulsed radiofrequency (PRF) of the dorsal root ganglion, lumbar sympathetic ganglion block, sacroiliac joint injection, intradiscal injection, facet rhizotomy, percutaneous epidural adhesiolysis, and spinal cord stimulation, over two years (*p*=0.36).

**Figure 2:**
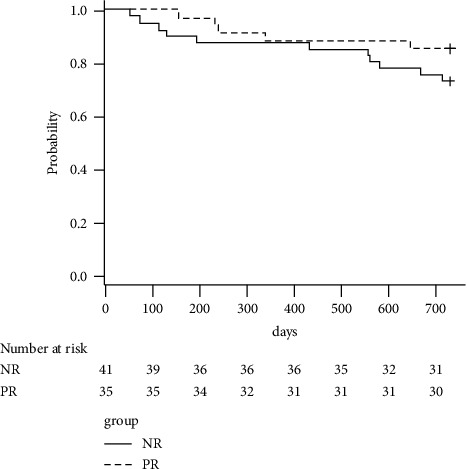
Kaplan–Meier curves. PR: positive response group, NR: no response group.

The number of patients who underwent surgery within one year was four and five in the PR and NR groups, respectively, and no significant difference was noted between the two groups (*p*=0.60). The overall incidence of surgery was 11.8% at one year after the first TFESI. One patient in the NR group had progressive lower extremity muscle weakness, and the other eight patients requested surgery. Surgery was performed within two years in five patients in the PR group and eleven in the NR group. No significant difference was found in the incidence of surgery over two years between the two groups (*p*=0.26). The Kaplan–Meier curves are shown in Figure 2. The selection of surgical treatment in all six additional patients was based on patient preference.  Table 4 shows the details of the 16 patients who underwent surgery. Regarding the number of patients who had central stenosis, foraminal stenosis, or both, there was no difference between the PR and NR groups (Table 2). The overall incidence of surgery was 21.2% over two years. After adjusting for baseline NRS, the hazard ratio of the PR group to the NR group regarding the incidence of surgery within two years was 0.35 (95% confidence interval: 0.11–1.11, *p*=0.08).

EQ5D5L after one month was significantly higher in the PR group than in the NR group (*p* < 0.01). Moreover, significantly more patients in the PR group had increased EQ5D5L by 0.1 or more from the initial visit (*p*=0.01).

No major complications related to TFESI were observed, including spinal cord infarction, nerve injury, epidural hematoma, or epidural abscess.

## 4. Discussion

TFESI is a minimally invasive intervention that is often preferred by clinicians for treating lumbar radiculopathy [13]. In clinical practice, owing to its relatively low invasiveness, patients with lumbar radiculopathy frequently attempt treatment with TFESI before considering surgery, and interventional treatments including TFESI are often undergone repeatedly. According to a systematic review assessing the effect of epidural injections for lumbar radiculopathy, less than half of the studies were followed up for one year, and only five out of 40 studies assessed the results at two years [14]. Therefore, we believe that an examination of the long-term effects of TFESI is needed.

One prospective cohort study showed that younger age and working full-time were predictors of a better outcome of chronic lumbar radiculopathy [15]. In this study, there were no significant differences in patient background between the two groups.

Although there was no statistically significant difference between the two groups regarding the rate of surgery (*p*=0.26), this rate was nearly twice as high in the NR group (NR group, 26.8% and PR group, 14.3%). Moreover, among patients who opted for surgery, patients in the PR group mostly had surgery within the first year, whereas those in the NR group who opted for surgery were evenly distributed between the first and second years of follow-up. Therefore, if we examined the patients over a longer period, there is a possibility that the two groups could have had a significant difference in the proportion of surgery.

Although, at baseline, all patients preferred nonsurgical treatment in this study, more than one factor influencing patient selection for surgical treatment was identified. Regarding the reason for the decision to undergo surgery, Lurie et al. examined 740 patients diagnosed with intervertebral disc herniation and showed that patients preferring surgery were younger, had a lower level of education, and had higher levels of unemployment [12]. They also showed that the expectations of patients regarding improvement with nonoperative care were the strongest predictors of preference.

Regarding EQ5D5L, no significant difference was found between the two groups at baseline, but a significant difference was noted after one month, and significantly more patients in the PR group showed an increase of 0.1 or greater (Table 1). These findings indicate that pain relief is associated with an improved health-related QOL.

In this study, all participants were considered operative candidates by spine surgeons and may have transitioned to surgery if they did not have an opportunity to consult a pain specialist. A prospective randomized double-blind controlled study evaluated the efficacy of periradicular infiltration with corticosteroids for radicular pain due to lumbar disc herniation or lumbar spinal stenosis [16]. In their study, the overall rate of surgery was 18% at a minimum of one year after the injection. In addition, an observational study that evaluated the use of TFESI as an alternative to surgery in patients with lumbar spinal stenosis showed that the overall rate of surgery was 32% at the two-year follow-up [17]. In this study, approximately 50% of the patients in both groups were diagnosed with spinal canal stenosis. The rates of surgery were 13.2% at one year after the first TFESI and 21.1% at two years after the first TFESI, which are lower than those reported in previous studies. Subsequently, 78.9% (60/76) of the patients avoided surgery for two years. A systematic review assessing the efficacy of TFESI showed that repeated TFESIs yield better outcomes [18]. Our results suggest that repeated interventions help patients avoid surgery.

The epidural steroid dose used in our study (1.65 mg dexamethasone) is lower than those in the abovementioned studies [3–5]. A randomized double-blind trial examined the effective steroid dose in TFESI for pain reduction in patients with lumbosacral radiculopathy [19]. In this study, 160 participants were randomly assigned to four groups (epidural injections of either 5, 10, 20, or 40 mg of triamcinolone). All groups showed improvements in verbal NRS scores at one week after the second TFESI. However, the group treated with 5 mg of triamcinolone showed less improvement in participant satisfaction compared to the other groups. The authors concluded that a dose of at least 10 mg of triamcinolone is sufficient to provide significant pain relief. However, particulate steroids such as triamcinolone have a potential risk of spinal cord infarction due to embolism of radicular vessels. Moreover, one observational study showed the intermediate-term safety of repeated transforaminal epidural injections with 1.65 mg of dexamethasone regarding effects on glucose profile and pituitary-adrenal axis functions [20]. Thus, in Japan, we always use 1.65 mg dexamethasone which has a strength approximately equivalent to 10 mg triamcinolone for epidural injections to avoid potential adverse effects from an excess of exogenous corticosteroids.

According to a randomized control study, PRF treatment of the dorsal root ganglion shows longer-term pain relief and improvement than TFESI [21]. In this study, only 25.8% of patients (16/62) had undergone PRF as an additional treatment. Proactive implementation of PRF may help enhance the surgery-avoidance effect.

### 4.1. Limitations

This study has several limitations. First, the sample size was small. The incidence of surgery was only 26.8% in the NR group. Based on this result, this study would have required a larger sample. Second, this was a single-center study. Although the need for surgery and the timing of surgery have been extensively studied, controversies remain [22]. In the present study, patients who were determined by a spine surgeon to be indicated for surgery were referred to the pain center, and a pain specialist determined their suitability for enrolment in the study. Matching the criteria of patients eligible for surgical treatment is difficult for spine surgeons; therefore, the results of this study cannot be generalized. Third, this study did not use blinding. The attending physician provided both therapy and evaluation. If an independent person evaluated the efficacy of TFESI, the results might be different. Fourth, the therapies administered after the first month were not uniform. The type and frequency of intervention were left to the discretion of the attending physician. Pharmacotherapy, patient education, and exercise therapy were administered. The results may vary depending on the aforementioned differences because the efficacy of education and exercise therapy for patients with chronic pain has been demonstrated [23]. Fifth, there were issues regarding bias. All patients in this study requested nerve block treatment before surgery. In other words, they did not want to undergo surgery. This bias might have led to the lower surgery rate. Moreover, studies conducted by interventionists tended to report positive results at a rate three times higher than those conducted by noninterventionists [24]. This study was jointly conducted by spine surgeons and pain specialists so that patients could undergo surgery at any time. However, many patients visited pain specialists more often than spine surgeons, and pain specialists may have unknowingly influenced the patients to avoid surgery. Finally, the reason for all patients who chose surgery was not determined. More than half of the patients (9/16) were not satisfied with the effects of interventional treatment. The preference for surgery may be influenced not only by pain intensity but also by other factors. Despite these limitations, the strength of this study is that it is prospective and reflects the typical real-world referral pattern of patients to pain centers for interventional treatment in cases of refractory lumbar radiculopathy.

## 5. Conclusions

A positive response to TFESI did not identify patients who can avoid surgery for two years in a clinical setting; however, the incidence of surgery was nearly twice as high in the NR group compared to the PR group. Repeating the intervention has 78.9% of surgery-avoiding effects for two years. Interventional treatments are worth attempting before surgery. Future studies with larger sample sizes, longer-term follow-ups, and examinations of the factors related to patient preference and the reasons they chose surgery are warranted.

## Figures and Tables

**Table 1:** Patients' demographics and results.

	PR group (*n* = 35)	NR group (*n* = 41)	*p* values
Age (years)	73.0 (11.0)	72.0 (10.0)	0.53
Female	17 (48.6)	23 (56.1)	0.65
Employment status			0.13
Without occupation	2 (5.7)	0 (0)	
Holding a job	17 (48.6)	27 (65.9)	
Old age pensioner	16 (45.7)	14 (34.1)	
Primary disease			0.87
Central stenosis	4 (11.4)	5 (12.2)	
Foraminal stenosis	11 (31.4)	15 (36.6)	
Both	20 (57.1)	21 (51.2)	
Duration of illness (months)	6.0 (16.5)	6.0 (11.0)	0.46
Treatment before baseline			
Epidural injection	12 (34.3)	10 (24.4)	0.45
iv steroid	1 (2.9)	4 (9.8)	0.37
NRS at baseline	7.5 (1.65)	6.5 (3.0)	<0.01
EQ5D5L at baseline	0.58 (0.23)	0.52 (0.24)	0.52
HADS anxiety score	6.0 (5.0)	7.0 (7.0)	0.45
HADS depression score	6.0 (5.5)	6.0 (4.0)	0.91
PCS	35.0 (15.0)	36.0 (6.0)	0.53
Surgery within 1 year	4 (11.4)	6 (14.6)	0.75
Surgery within 2 years	5 (14.3)	11 (26.8)	0.26
NRS at 1 month	3.9 (2.3)	7.0 (2.7)	<0.01
EQ5D5L at 1 month	0.71 (0.23)	0.60 (0.28)	<0.01
More than 0.1 points improvement of EQ5D5L at 1 month	19 (54.3)	10 (24.4)	0.01

Median (interquartile range) or number (%), PR group: positive response group, NR group: no response group, NRS: Numerical Rating Scale, EQ5D5L: EuroQOL 5-Dimension 5-Level, HADS: Hospital Anxiety and Depression Scale, PCS: Pain Catastrophizing Scale, iv steroid: intravenous steroid administration.

**Table 2:** Overview of the primary diseases, treatment levels, and percentages of patients who underwent surgery.

Primary disease	PR group (*n* = 35)			NR group (*n* = 41)		
	Central stenosis (*n* = 4)	Foraminal stenosis (*n* = 11)	Both (*n* = 20)	Central stenosis (*n* = 5)	Foraminal stenosis (*n* = 15)	Both (*n* = 21)
TFESI						
One side	4	11	17	4	13	19
Both sides	0	0	3	1	2	2
Surgery	1	1	3	3	3	5

PR group: positive response group, NR group: no response group, TFESI: transforaminal epidural steroid injection.

**Table 3:** Details of the interventions.

	PR group (*n* = 35)	NR group (*n* = 41)	*p* values
Continuation of visiting our pain center for 1 year	18 (51.4)	29 (70.7)	0.10
Continuation of visiting our pain center for 2 years	10 (28.6)	21 (51.2)	0.25
Total times of interventions for 2 years	6.0 (1.0–37.0)	13.0 (1.0–42.0)	0.34
Total times of steroid (1.65 mg of dexamethasone) use for 2 years	2.0 (1.0–8.0)	3.0 (1.0–14.0)	0.01
Number of patients who underwent interventions only for the first month	8 (22.9)	6 (14.6)	0.36
Number of patients who underwent interventions for more than one month	27 (77.1)	35 (85.4)	0.36
EI (IL, TF, and Cau)	13 (48.1)	14 (40.0)	
DRG PRF	7 (25.9)	9 (25.7)	
LSBG	6 (22.2)	15 (42.9)	
SIJ	3 (11.1)	3 (8.6)	
IDI	2 (7.4)	1 (2.9)	
PEA	5 (18.5)	6 (17.1)	
FR	3 (11.1)	4 (11.1)	
SCS	0 (0)	1 (2.9)	

Median (minimum-maximum) or number (%), PR group: positive response group, NR group: no response group, EI: epidural injection, IL: interlaminar approach, TF: transforaminal approach, Cau: caudal approach, DRG PRF: pulsed radiofrequency of the dorsal root ganglion, LSBG: lumbar sympathetic ganglion block, SIJ: sacroiliac joint injection, IDI: intradiscal injection, PEA: percutaneous epidural adhesiolysis, FR: facet rhizotomy, SCS: spinal cord stimulation.

**Table 4:** Details of the patients who chose surgery.

Case no.	Group	Age (years)	Sex	HADS (A)	HADS (D)	PCS	Disease duration (months)	Duration before operation (months)	Baseline NRS	NRS at 1 month	Reason for choosing surgery
1	PR	25	m	9	10	39	1.25	8	6.4	3.3	Family's recommendation
2	PR	74	m	4	6	23	6	11	8.0	3.0	Dissatisfaction
3	PR	75	f	12	9	35	7	22	8.0	6.0	Pain aggravation
4	PR	81	f	8	1	47	3	5	10.0	8.0	Dissatisfaction
5	PR	83	m	4	5	42	8	8	7.5	3.6	Dissatisfaction
6	NR	59	f	11	3	30	9	2	4.0	7.0	Dissatisfaction
7	NR	70	m	3	3	26	3	5	7.0	6.0	Dissatisfaction
8	NR	65	f	2	8	41	6	6	7.5	7.0	Muscle weakness
9	NR	53	f	7	3	35	12	14	9.0	8.0	Dissatisfaction
10	NR	75	f	4	6	24	60	23	6.4	6.5	Pain aggravation
11	NR	68	m	11	6	50	15	4	5.0	7.0	Dissatisfaction
12	NR	68	f	8	12	41	3	20	7.5	8.0	Unknown
13	NR	70	f	9	12	33	5	20	7.0	7.0	Pain aggravation
14	NR	80	m	14	6	43	4	18	8.0	8.0	Unknown
15	NR	74	m	2	7	17	240	18	6.5	7.0	Dissatisfaction
16	NR	75	f	5	1	24	1	3	9.0	8.0	Dissatisfaction

All cases except case 8 preferred surgery. Case 8 had progressive muscle weakness in the leg. TFESI: transforaminal epidural steroid injection; Group: PR: positive response to TFESI, NR: no response to TFESI; sex: f: female, m: male; NRS: Numerical Rating Scale, EQ: EuroQOL 5-Dimension 5-Level, HADS (A): anxiety score of the hospital anxiety and depression scale, HADS (D): depression score of the Hospital Anxiety and Depression Scale, PCS: Pain Catastrophizing Scale; primary disease: SS: spinal canal stenosis, DH: disc herniation, LS: lumbar spondylolisthesis; treatment before baseline: epi: epidural injection, iv: intravenous steroid administration, med: oral medication.

## Data Availability

The datasets generated and/or analyzed during the current study are available from the corresponding author on reasonable request.
